# Significant Correlation Between Cutaneous Abundance of *Streptococcus* and Psoriasis Severity in Patients with FBXL19 Gene Variants

**DOI:** 10.2340/actadv.v104.34892

**Published:** 2024-06-19

**Authors:** Malin ASSARSSON, Jan SÖDERMAN, Oliver SEIFERT

**Affiliations:** 1 Department of Biomedical and Clinical Sciences, Faculty of Health Sciences, Linköping University, Linköping, Sweden; 2 Laboratory Medicine, Region Jönköping County, Jönköping, Sweden; 3 Division of Dermatology and Venereology, Region Jönköping County, Jönköping, Sweden

**Keywords:** microbiome, psoriasis, SNPs

## Abstract

Psoriasis results from both genetic predisposition and environmental triggers, such as *Streptococcal* infections. This study aimed to explore the correlation between the abundance of the *Streptococcus* genus on the skin and psoriasis severity in individuals carrying specific psoriasis-associated genetic variants. Studying 39 chronic plaque psoriasis patients, the elbow skin microbiome and 49 psoriasis-related single nucleotide polymorphisms (SNPs) were analysed using a MiSeq instrument for 16S rDNA sequencing, and CLC Genomic Workbench for processing and analysis. Through multivariate linear regression analysis, a positive correlation was found between *Streptococcus* genus abundance and psoriasis severity in patients with certain FBXL19 gene-related heterozygous SNPs (rs12924903, rs10782001, rs12445568). Conversely, a negative association was observed in patients with homozygous genotypes. Moreover, we identified an association between *Streptococcus* abundance and psoriasis severity in patients with genetic variants related to IL-22, ERAP1, NOS2, and ILF3. This is the first study highlighting a positive association between *Streptococcus* skin colonization and psoriasis severity in patients with heterozygous genotypes within the FBXL19 gene region. FXBL19 targets the IL-33/IL1RL1 axis, crucial in infectious diseases and innate immunity promotion. These novel results suggests an intricate interaction among host genetics, *Streptococcus* skin colonization, and psoriasis inflammation, offering potential avenues for novel treatment approaches.

SIGNIFICANCE

In our study, we discovered a correlation between the prevalence of *Streptococcus* on the skin and the severity of psoriasis in patients with specific genetic variants linked to the FBXL19 gene. Our results suggest that bacterial colonisation affect psoriasis severity differently, based on a patient’s genetic makeup. This insight might pave the way for tailored treatments targeting particular genetic variants, potentially enhancing their effectiveness in managing psoriasis.

The common immune-mediated inflammatory disease psoriasis has an estimated prevalence of 2–3% (1). Patients with psoriasis have a significantly increased risk of comorbidity, such as heart disease, inflammatory bowel disease (IBD), and obesity (2, 3). It is well known that psoriasis can be both triggered and exacerbated by bacterial colonization of various parts of the body (4, 5). More recently, the microbiome of different parts of the body, particularly the gut, has been implicated in a variety of other inflammatory and systemic autoimmune diseases such as diabetes mellitus, rheumatoid arthritis, and IBD (6–8). Dysbiosis of the skin in genetically predisposed individuals is associated with activation of the innate immune system, inducing an adaptive immune response that can lead to psoriasis (9, 10). Studies have been conducted to understand the skin microbiota in psoriasis, but results have been inconsistent. Some studies have demonstrated a more diverse microbiome in psoriatic plaques compared with unaffected skin sites or in skin of healthy controls (10, 11), while in other studies a trend towards reduced alpha diversity in lesional psoriatic skin has been noted (12–14). Our group showed that treatment of psoriasis with narrowband ultraviolet B affects the microbiome (15) and that the relative abundance of several genera in the skin correlated with psoriasis severity (16). These studies indicate that the skin bacterial communities in psoriasis may be altered in a significant manner, but further exploration is needed to conclude how this directly impacts the pathogenesis of psoriasis.

Various large-scale genome-wide association studies have identified potential risk factors and susceptibility loci for psoriasis. These loci contain genes involved in various processes such as antigen presentation, Th1 cell differentiation, nuclear factor kappa B (NF-ĸB) signalling, interferon signalling, and keratinocyte proliferation (17), providing genetic evidence linking immune dysfunction to psoriasis predisposition (18–20). MHC class 1 is the genetic locus with the strongest association to psoriasis susceptibility (19, 20).

Streptococcal infections of the upper respiratory tract are the most well-known bacterial trigger of psoriasis, which induces mainly guttate psoriasis (21–24). *Streptococcus pyogenes* extract has been shown to induce a preferential Th17 response in patients with psoriasis (25), suggesting that *Streptococcal* infection is directly involved in the pathogenesis of psoriasis. In mice, repeat-ed infection with *group A Streptococcus* led to exacerbation of imiquimod-mediated psoriatic skin lesions (26). One possible mechanism for how *Streptococcal* infection triggers psoriasis involves the priming and selection of tonsillar T-cells by the bacteria in the pharynx, followed by their migration into the skin and subsequent reactivation and clonal expansion (27). Tonsils from patients with psoriasis have been shown to have a higher frequency of cutaneous lymphocyte-associated antigen CD4+ and CD8+ T-cells and a higher frequency of tonsil T-cells expressing the IL-23 receptor, suggesting a dysregulated immune response in the tonsils of psoriatic patients (28). Another possible mechanism involves *Streptococcal* superantigens, which can bypass normal immunological pathways and stimulate the immune system powerfully. These superantigens include pyrogenic exotoxin and M protein (29). However, a recent Cochrane review (30) found that there is currently insufficient evidence to establish a link between psoriasis and *Streptococcal* infection.

As of our current understanding, there are no recent research data available regarding the correlation between genetic variations, psoriasis, and the abundance of *Streptococcus* on the skin. Consequently, the objective of this study is to investigate the potential correlation between the abundance of the *Streptococcus* genus on the skin and the severity of psoriasis in individuals with specific genetic variants known to be associated with psoriasis.

## MATERIALS AND METHODS

### Study population

This study included a total of 50 patients diagnosed with plaque-type psoriasis. However, DNA-purification protocols yielded insufficient DNA in 3 cases and sequencing generated too few reads in 8 cases, excluding these individuals from the study. Therefore, the final data analysis included 39 participants. None of the patients had used topical antiseptics, oral antibiotics, systemic anti-inflammatory, or immune-modulating treatment for at least 3 months prior to entering the study, and had not used topical corticosteroids on the target lesion 2 weeks prior to the study. Participants who were pregnant, had undergone tanning or intensive sun exposure in the past 2 weeks, were under the age of 18 years, had a known malignancy, psoriatic arthritis or other systemic inflammatory condition, or showed symptoms of infection at the time of sample collection were excluded from the study. All participants lived in the same geographical region of Sweden to reduce the impact of environmental and dietary factors. Written informed consent, including consent to publication of their case details, was obtained from all participants and the study was approved by the ethics committee of Linköping University, Linköping, Sweden (approval number 2014/179-31). The participants’ gender, age, height, weight, current diseases and medications, smoking and alcohol habits, and family history of psoriasis were recorded. The severity of psoriasis was assessed using the Psoriasis Area and Severity Index (PASI) by a trained dermatology nurse. Of the 39 patients included in the final analysis, 8 had mild (PASI < 3), 23 had moderate (PASI 3–9.9) and 8 had severe psoriasis (PASI ≥ 10). Patients’ demographic and clinical characteristics are given in **Table I**. The participants’ other diseases and medications are listed in Table SI.

**Table I:** Patients’ demographic and clinical characteristics

Number of subjects	39
Age, years, mean ± SD (range)	54.1 ± 14.9 (24–76)
Male:female ratio, *n*	20:19
Body mass index, mean ± SD (range)	26.8 ± 3.2 (20.8–33.0)
PASI, mean ± SD (range)	6.2 ± 4.6 (0.5–25)
Family history of psoriasis, %	66.7
Smoker, %	10.3
Alcohol intake ≥ 2 times/month, %	43.6

SD: standard deviation; PASI: Psoriasis Area and Severity Index.

### Sample collection, preparation, sequencing, and data analysis

Samples were taken from lesional elbow skin of patients with psoriasis by swabbing a 4x4 cm area with a flocked swab soaked in 1 mL liquid Amies (ESwab™, Copan Diagnostics Inc., Murrieta, CA, USA). Because colonization of bacteria in the skin depends on the skin site (31), all samples were taken from the elbow area. The samples were stored at –20°C, for up to 3 months, until DNA isolation. DNA extraction, sequencing, sequence processing, classification, and data analysis has previously been described (16). In short, a MiSeq instrument (Illumina, CA, USA) was used for 16S rDNA sequencing, and files in the FASTQ format were imported as paired-end reads into the CLC Genomic Workbench (http://www.clcbio.com Version 20.0) for processing and analysis. A reference-based approach (SILVA 16S v.132) was used for clustering, with a similarity threshold of 97%, and chimeric sequences were removed.

Venous blood samples were collected in EDTA from all included patients and stored at –80°C prior to DNA extraction.

### Single nucleotide polymorphisms (SNP) selection

A systematic literature search (PubMed) was conducted to identify 56 single-nucleotide polymorphisms (SNPs). Genetic variants in susceptibility loci that have previously been associated with increased risk of psoriasis were included as well as SNPs in genes coding for cytokines involved in psoriasis pathogenesis and innate immune response (18–20, 32–40) (Table SII). When processing the data, the GWAS catalogue (https://www.ebi.ac.uk/gwas, accessed 17 May 2023), in addition to the literature search, was used to further investigate genes of interest in the examined susceptibility loci.

### DNA preparation

Genomic DNA was extracted from blood collected in EDTA using MAGAttract DNA Blood Mini M48 Kit (Qiagen, Hilden, Germany) and DNA concentrations were determined by NanoDrop ND-1000 (Thermo Fisher Scientific, Wilmington, DE, USA). Purified DNA was stored at –20°C.

### Genotype assessment

Genotyping assays for 56 selected SNPs were designed, validated, and analysed by the Mutation Analysis Core Facility (MAF) at the Karolinska University Hospital (Huddinge, Sweden; https://www.maf.ki.se/) using the MassARRAY system from Agena Bioscience (San Diego, CA, USA). Assays were validated using human DNA samples from the CEU population, for which genotype data generated by the International HapMap Consortium is available. Reproducibility of the genotyping assays was ensured by repeated analysis on a number of samples. Further quality controls included 12 negative controls and 12 positive controls (CEU samples) on each 384 well plate, and analysed samples and SNPs were investigated for deviations from the Hardy–Weinberg equilibrium. Seven out of 56 SNP assays failed due to technical problems. Thus, 49 SNPs were included in the statistical analysis (Table SII).

### Statistical analysis

A multivariate linear regression analysis was conducted using the IBM SPSS statistical software version 27 for Windows (IBM Corp ,Armonk, NY, USA) in order to analyse the association between PASI and gender, age, BMI, smoking, alcohol, relative abundance of *Streptococcus* genus, and SNPs. One model was made for each SNP.

Microbiome data are compositional data (41) and hence, a centred log-ratio (CLR) transformation was performed prior to the extraction of *Streptococcal* abundance data (strepCLR). Furthermore, PASI values were transformed using the natural logarithmic to mitigate heteroscedasticity, henceforth referred to as transformed PASI.

*P*-values were adjusted for multiple testing using the Bonferroni procedure, and a Bonferroni adjusted i-value of < 0.05 was considered significant.

## RESULTS

Of the 49 analysed SNPs (Table SII), the multiple regression model found statistically significant relations between transformed PASI values and the transformed abundance of *Streptococcus* on the skin, SNP genotypes, and their interactions for seven SNPs (**Table II**; for the complete set of results, see Table SIII). Age, BMI, gender, alcohol, and smoking variables did not contribute significantly to the models (*p* > 0.05).

**Table II:** Significant results from multiple regression analyses, using transformed Psoriasis Area and Severity Index (PASI) as a dependent variable

Parameter estimates	B	SE	95% Wald CI		Wald χ^2^	Sig.
			LL	UL		
**rs10782001**						
strepCLR	–0.60	0.18	–0.96	–0.25	10.46	*
GG/AA	42.21	11.45	19.76	64.66	13.58	*
GA/AA	–5.36	1.45	–8.20	–2.52	13.68	*
GA/GG	–47.57	11.79	–70.67	–24.47	16.29	**
GG/AA*strepCLR	–6.996	1.89	–10.70	–3.30	13.71	*
GA/AA*strepCLR	0.82	0.22	0.39	1.25	13.99	**
GA/GG*strepCLR	7.84	1.92	4.07	11.59	16.67	**
**rs12924903**						
strepCLR	–7.60	1.89	–11.29	–3.89	16.17	**
GG/AA	–42.21	11.45	–64.66	–19.76	13.58	*
GA/AA	–47.57	11.79	–70.67	–24.47	16.29	**
GA/GG	–5.36	1.45	–8.20	–2.52	13.68	*
GG/AA*strepCLR	7.00	1.89	3.30	10.70	13.71	*
GA/AA*strepCLR	7.82	1.92	4.05	11.59	16.53	**
GA/GG*strepCLR	0.82	0.22	0.40	1.25	13.99	**
**rs12445568**						
strepCLR	–0.64	0.18	–0.99	–0.285	12.59	*
TC/TT	–5.04	1.4	–7.79	–2.29	12.94	*
TC/TT*strepCLR	0.80	0.21	0.39	1.20	14.56	**
**rs2046068**						
strepCLR	1.04	0.17	0.71	1.37	38.39	***
TT/GG	9.09	1.96	5.26	12.93	21.58	***
GT/GG	8.47	1.38	5.76	11.18	37.51	***
TT/GG*strepCLR	–1.19	0.26	–1.71	–0.69	21.17	***
GT/GG*strepCLR	–1.04	0.16	–1.35	–0.73	44.27	***
**rs27432**						
strepCLR	–2.03	0.39	–2.81	–1.26	26.39	***
GG/AA	–13.94	2.76	–19.35	–8.53	25.53	***
GA/AA	–11.59	2.86	–17.21	–5.99	16.39	**
GG/AA*strepCLR	2.31	0.39	1.53	3.08	34.24	***
GA/AA*strepCLR	1.94	0.39	1.16	2.71	23.95	***
**rs4795067**						
AG/AA	–6.81	1.75	–10.25	–3.37	15.07	**
AG/AA*strepCLR	0.99	0.25	0.49	1.49	15.56	**
**rs892085**						
GA/AA	5.37	1.48	2.46	8.27	13.13	*
GA/AA*strepCLR	–0.85	0.22	–1.28	–0.43	15.80	**

All variables were included in the model by selecting the default method (“Enter”) for multivariate linear regression analysis using the SPSS statistical software. strepCLR: centred log-ratio transformation streptococcus abundance; B: unstandardized regression coefficient; CI: confidence interval; LL: lower limit; UL: upper limit; SE: standard error of the coefficient; ns = not significant; ref = reference alleles used;

* *p* < 0.05,

** *p* < 0.01,

*** *p* < 0.001 (Bonferroni adjusted for multiple comparison).

**Fig. 1:**
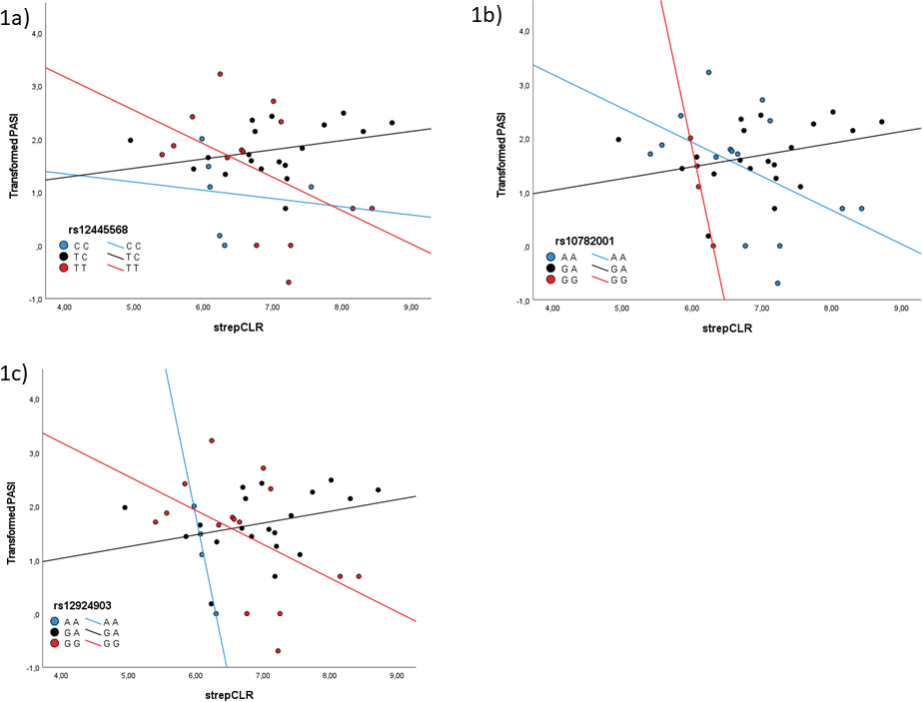
Scatterplot visualising transformed Psoriasis Area and Severity Index (PASI) and centred log-ratio transformed *Streptococcal* abundance (strepCLR) for different genotypes of (a) rs12445568, (b) rs10782001, and (c) rs12924903.

Three of the significant SNPs (rs12445568, rs10782001, and rs12924903) were in close proximity to the FBXL19 gene, and for all 3 SNPs, the interaction of *Streptococcal* abundance and genotype was significantly associated with transformed PASI, where the heterozygous geno-types had a positive association and the homozygous genotypes had a negative association (Table II), as visualized in **Fig. 1** a–c. For rs10782001 and rs12924903, there was also a significant association between the abundance of *Streptococcus* and transformed PASI and between genotypes and transformed PASI (Table II).

**Fig. 2:**
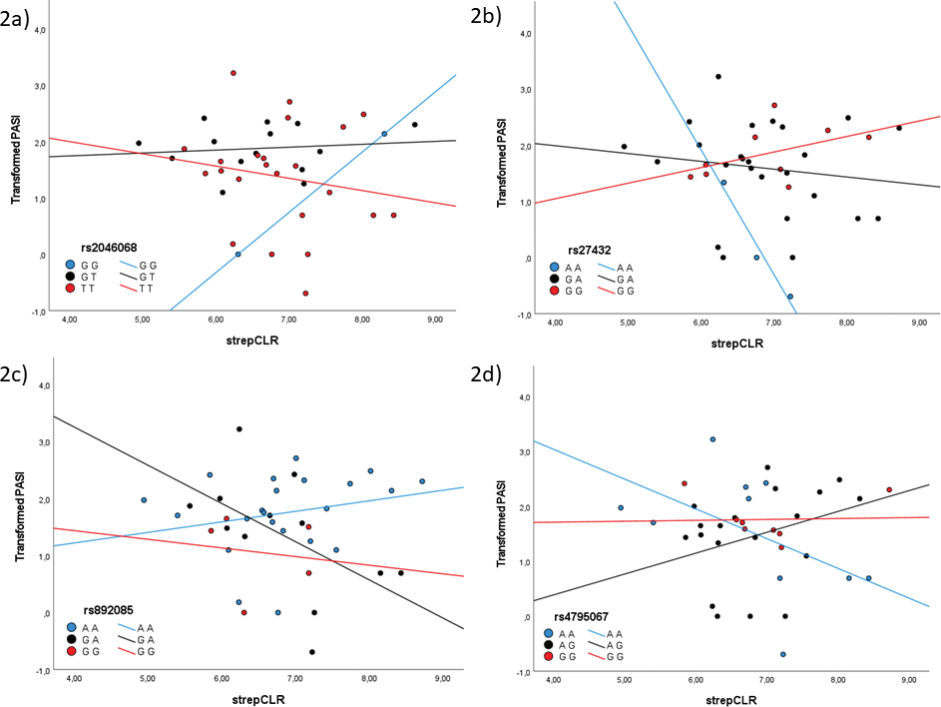
Scatterplot visualising transformed Psoriasis Area and Severity Index (PASI) and centred log-ratio transformed *Streptococcal* abundance (strepCLR) for different genotypes of (a) rs2046068, (b) rs27432, (c) rs892085, and (d) rs4795067.

For the SNPs rs2046068 and rs27432, the interaction of *Streptococcal* abundance and genotype was significantly associated with transformed PASI, as shown in **Fig. 2** a–b. There was also a significant association between the abundance of *Streptococcus* and transformed PASI and between genotype and transformed PASI (Table II).

For the SNPs rs892085 and rs4795067, the interaction of *Streptococcal* abundance and genotype was significantly associated with transformed PASI, presented in **Fig. 2** c–d. There was also a significant association between genotype and transformed PASI (Table II).

## DISCUSSION

In our study, we observed a significant correlation between the severity of psoriasis and the interaction between genotypes and the abundance of *Streptococcus* genus on the skin, for the SNPs rs12445568, rs10782001, and rs12924903, all related to the FBXL19 gene, and for the SNPs rs2046068 (IL22), rs27432 (ERAP1), rs892085 (ILF3), and rs4795067 (NOS2). In all 3 SNPs relating to the FBXL19 gene, the interaction between the abundance of *Streptococcus* on the skin and genotype showed a significant association with disease severity (PASI), with a positive association between *Streptococcal* levels and disease severity for the heterozygous genotypes and a negative association for the homozygous genotypes.

The F-box and leucine-rich repeat protein 19 (FBXL19) gene encodes a member of the Skp1-cullin-F-box family of E3 ubiquitin ligases (42). FBXL19 targets IL1RL1 and Rac1 for their polyubiquitination and proteosomal degradation (43). It is also likely that FBXL19 functions as an antagonist of RhoA, influencing several processes that regulate cell growth and cell motility (44). Moreover, FBXL19 is structurally related to FBXL11, which has been shown to inhibit NF-κB activity by lysine demethylation (45). Jumonji C domains are known to be required for demethylase activity, and while FBXL11 contains these, FBXL19 does not (46), leading to speculation that FBXL19 acts as a dominant negative inhibitor of demethylase activity, thereby activating NF-κB(19).

The FBXL19-related SNPs rs12924903, rs10782001, and rs12445568 have all previously been linked to psoriasis, for which the risk allele is A, G, and C, respectively (19, 20). Patients carrying the FBXL19 rs10782001-GG genotype have been shown to be at a higher risk of developing paradoxical psoriasis when treated with anti-TNF drugs (47). FBXL19 has been demonstrated to target the IL-33-tumorigenicity 2 IL1RL1 axis, selectively mediating the ubiquitination and degradation of IL1RL1 to limit IL-33-induced pulmonary inflammation (43). The IL-33/IL1RL1 axis plays diverse roles in various infectious diseases, and studies have suggested that it confers a protective effect against *Group A Streptococcus* infection by enhancing innate immunity (48). In our study, the interaction between genotypes and the abundance of *Streptococcus* on the skin showed a significant association with severity of psoriasis for these 3 SNPs. Although heterozygous genotypes revealed a notable positive correlation between abundance of *Streptococcus* and severity of psoriasis, it was rather unexpected that homozygosity for both reference alleles and risk alleles exhibited negative correlations. Given the suspicion that *Streptococcal* infections serve as triggers for psoriasis, one would anticipate positive correlations among psoriasis patients with homozygous risk genotypes. Nevertheless, the correlations observed in relation to homozygous risk genotypes were based on fewer samples, making them less reliable compared with both heterozygous individuals and homozygosity in respect of the reference alleles, which displayed the expected correlations.

Rs2046068 is located in an intron of the IL-22 gene. Previous investigations have shown that this polymorphism is not associated with chronic plaque psoriasis (39). However, IL-22 has been found to regulate the expressions of genes involved in antimicrobial proteins, differentiation-associated proteins, and mobility- and migration-regulating proteins in human keratinocytes, all of which are functions altered in psoriatic keratinocytes (49). We have previously found IL-22 levels to be lower in lesional skin than non-lesional skin in patients with psoriasis and that the levels were unaffected by narrowband ultraviolet B treatment (50). IL-22 levels in serum have been shown to correlate with PASI of psoriasis patients, and psoriasis patients colonized with toxigenic strains of *Staphylococcus aureus* had significantly higher levels of IL-22 in serum compared with those colonized with non-toxigenic strains (51). It can be speculated that colonization of the skin with *Streptococcus* in combination with genetic variations in IL-22 affects psoriasis severity, considering that the interaction between genotypes of rs2046068 and the abundance of *Staphylococcus* on the skin correlated with the severity of psoriasis.

Rs27432 is located in an intron of the endoplasmic reticulum aminopeptidase 1 (ERAP1) gene, which encodes a zinc metalloprotease aminopeptidase involved in trimming peptides for MHC class I presentation (52). ERAP1 polymorphisms have been linked to several autoimmune diseases, including psoriasis, with the risk allele A (20, 53, 54). Psoriatic lesions have been shown to have decreased ERAP1 and increased ERAP2 expression. It has been suggested that an individual’s ERAP variant must generate autoantigenic peptides capable of stimulating pathogenic autoreactive T cells, in addition to the ratio of ERAP2 to ERAP1 favouring autoantigen processing for autoimmunity to occur (55). An ERAP1 risk haplotype may increase the production of an autoantigenic peptide, leading to its presentation by HLA-C*06:02 and the activation of CD8+ T cell, which triggers autoimmune disease (56). In our study, the AA genotype differed significantly from both the GA and GG genotype in correlation between abundance of *Streptococcus* on the skin and severity of psoriasis.

Rs892085 is located in an intron of the QTRT1 gene, in close proximity to the ILF3 and CARM1 genes, and has been associated with psoriasis, with the risk allele A (20). Coactivator-associated arginine methyltransferase 1 (CARM1) is a transcriptional coactivator but is also believed to have functions including autophagy, metabolism, and pre-mRNA splicing and export (57). Interleukin enhancer binding factor 3 (ILF3) encodes a protein that interacts with other proteins, dsRNAs, small noncoding RNAs, and mRNAs to stabilize mRNAs and regulate gene expression. It is a subunit of the nuclear factor of activated T cells (NFAT), which is a transcription factor required for expression of IL-2 (20). ILF3 has been identified as a negative regulator of innate immune responses and dendritic cell maturation (58), which could explain our results showing an interaction between alleles of rs892085 and the abundance of *Streptococcus* on the skin correlated with psoriasis severity.

Rs4795067 is a polymorphism located in an intron of the nitric oxide synthase 2 (NOS2) gene, which is associated with psoriasis, with the risk allele G (59, 60). In murine macrophages, NOS2 expression and the production of nitric oxide have been shown to be induced after stimulation with lipopolysaccharide (LPS), suggesting that T-lymphocyte-mediated activation of macrophages is a potent stimulus for nitrate biosynthesis (61). Nitric oxide is believed to be particularly important in macrophages during infection with intracellular pathogens (62) and contributes to macrophage killing of *Streptococcus pneumonia* (63). Nitric oxide also mediates apoptosis through various mechanisms (64). Inhibition of NOS has been shown to exacerbate Group B *Streptococcus* sepsis and arthritis in mice (65). The link between NOS and the immune response to *Streptococcus* is intriguing, particularly given our discovery that the interaction between genotypes of rs4795067 and the abundance of *Streptococcus* on the skin is correlated with psoriasis severity.

### Limitations

There are some limitations to our study. We have a relatively small sample size, resulting in few data points for some of the alleles, and the majority of our participants had psoriasis of moderate severity. More participants with severe psoriasis might have led to different results. Considering the sample size and the number of predictor variables in our statistical model there is a risk of overfitting. However, as gender, age, smoking, BMI, and alcohol intake are known factors that can affect the severity of psoriasis (1), we considered these as important predictors and they were thus retained in the model regardless of their significance in the current sample of our exploratory study. Another limitation is that during participant recruitment, microbiome samples were stored at –20°C for up to 3 months prior to DNA isolation, instead of either snap frozen in liquid nitrogen and stored at –80°C or processed immediately. This may potentially influence the microbial composition and thereby the relative abundance of *Streptococcus* of the samples, thereby affecting the study’s results. Some strengths of our study are that all samples for microbiome analysis were taken from the same location and the results were adjusted for age, gender, alcohol, and smoking. The outcomes were subjected to rigorous adjustment for multiple testing using a stringent method (Bonferroni).

### Conclusion

Our results suggest that host genetic variations in combination with the skin’s microbiome have an impact on the severity of psoriasis, and the interaction between genetics, the immune system, and skin colonization with *Streptococcus* contributes to the complex nature of psoriasis. Genetic variations may impact the composition and behaviour of the skin microbiome, influencing how the immune system responds to bacteria and contributing to the development or exacerbation of psoriasis. This needs to be investigated further for any certain conclusions to be drawn, but understanding the genetic and microbial factors involved in psoriasis could lead to targeted treatments. There is a possibility that individuals with psoriasis carrying these genetic risk alleles may benefit from antibiotic treatment during psoriasis exacerbations.
